# Amperometric Miniaturised Portable Enzymatic Nanobiosensor for the Ultrasensitive Analysis of a Prostate Cancer Biomarker

**DOI:** 10.3390/jfb14030161

**Published:** 2023-03-17

**Authors:** Stefania Hroncekova, Lenka Lorencova, Tomas Bertok, Michal Hires, Eduard Jane, Marek Bučko, Peter Kasak, Jan Tkac

**Affiliations:** 1 Institute of Chemistry, Slovak Academy of Sciences, Dubravska Cesta 9, 845 38 Bratislava, Slovakia; 2 Center for Advanced Materials, Qatar University, Doha P.O. Box 2713, Qatar

**Keywords:** biomarker, early diagnostics, MXene, nanobiosensor, prostate cancer, sarcosine, sarcosine oxidase, screen-printed electrodes, urine

## Abstract

Screen-printing technology is a game changer in many fields including electrochemical biosensing. Two-dimensional nanomaterial MXene Ti_3_C_2_T_x_ was integrated as a nanoplatform to immobilise enzyme sarcosine oxidase (SOx) onto the interface of screen-printed carbon electrodes (SPCEs). A miniaturised, portable, and cost-effective nanobiosensor was constructed using chitosan as a biocompatible glue for the ultrasensitive detection of prostate cancer biomarker sarcosine. The fabricated device was characterised with energy-dispersive X-ray spectroscopy (EDX), electrochemical impedance spectroscopy (EIS) and cyclic voltammetry (CV). Sarcosine was detected indirectly via the amperometric detection of H_2_O_2_ formed during enzymatic reaction. The nanobiosensor could detect sarcosine down to 7.0 nM with a maximal peak current output at 4.10 ± 0.35 × 10^−5^ A using only 100 µL of a sample per measurement. The assay run in 100 μL of an electrolyte showed the first linear calibration curve in a concentration window of up to 5 μM with a slope of 2.86 μA·μM^−1^, and the second linear calibration curve in the range of 5–50 μM with a slope of 0.32 ± 0.01 μA·μM^−1^ (R^2^ = 0.992). The device provided a high recovery index of 92.5% when measuring an analyte spiked into artificial urine, and could be used for detection of sarcosine in urine for at least a period of 5 weeks after the preparation.

## 1. Introduction

Prostate cancer (PCa) is one of the leading global causes of cancer death and the most frequent type of cancer diagnosed for men in 112 countries. Around 650,000 new patients are diagnosed with prostate cancer each year, and the incidence continues to rise [1,2]. Among PCa risk factors, the following are at the top of the list: age, race, genetic predispositions, and family history. PCa is also linked with other factors, including eating habits (a high consumption of saturated fat of animal origin and red meat, and a low intake of fruits, vegetables, vitamins, and coffee), being overweight, physical inactivity, inflammation, hyperglycaemia, infections, and exposure to hazardous chemicals and radiation [3,4]. Male androgenic hormones also play key roles in the development and progression of PCa [5].

Most PCa types are slow-growing and often asymptomatic, or their symptoms are similar to those of benign prostate hyperplasia (BPH) and are, hence, underestimated [6,7]. Since PCa is often asymptomatic in its earlier stages, it is very difficult to diagnose it before it reaches advanced stages. Late stages of PCa are associated with frequent night urination (nocturia), pain and difficulties during urination (dysuria), and the presence of blood in urine (haematuria) [8,9]. PCa may also cause problems with sexual function and performance [10].

PCa is screened via the analysis of the serological level of the prostate-specific antigen (PSA). Initially, the US Food and Drug Administration (FDA) agency approved PSA tests together with a digital rectal examination (DRE) for PCa screening in 1994 [11]. A low level of PSA is present in healthy men, and an increased level is not only associated with PCa, but also with prostate enlargement, inflammation, and even cycling [7]. Hence, PSA is not a reliable PCa biomarker, with high false-negative and false-positive results due to the low specificity and sensitivity of the assay [7,8].

Since PSA is not able to provide reliable information as a biomarker for the diagnostics and screening of PCa, more specific biomarkers need to be studied and validated. Numerous types of biomolecules have been proposed as PCa biomarkers, including metabolites (i.e., sarcosine), proteins (i.e., prostate-specific antigen), RNAs (i.e., transmembrane protease serine 2-ETS-related gene fusion), and glycans [12,13,14,15].

Sarcosine is an intermediate metabolite involved in glycine synthesis and degradation. The first paper showing a link between changed sarcosine levels and cancer was published by A. Sreekumar and co-workers in 2009 [16]. Since this publication, there have been numerous studies that aimed to examine the association between sarcosine and PCa [17]. Elevated levels of sarcosine associated with prostate cancer can be found in prostate tissue, blood, and urine [18]. Significantly elevated levels of sarcosine are present in urine, and urine is the biofluid of choice since the analysis of biomarkers in urine is considered noninvasive [19].

The level of sarcosine in urine can vary from 20 nM to 5 µM [20]. The role of sarcosine in the onset and development of the disease is the stimulation of the growth of malignant and metastatic cells [21]. Various methods are available for the determination of sarcosine [18,22,23,24,25] that exhibit several drawbacks that can be addressed with biosensing devices that are simple, fast, inexpensive, and reliable. Electrochemical techniques coupled with SPCEs can offer additional benefits to users, including portability and miniaturisation [26,27,28]. The integration of such devices for “cancer-on-a-chip” assay platforms is an additional advantage for their use in many applied areas [29,30].

We have been witnessing a revolution in the integration of nanomaterials to design biosensors over the past few decades. MXenes, discovered in 2011, are one of the largest families of two-dimensional (2D) materials for a diverse range of applications, including biosensing due to their high electrical conductivity, large active area, hydrophilic nature, easily tuneable structure, excellent thermal stability, and large interlayer spacing [31,32,33,34].

**Figure 1:**
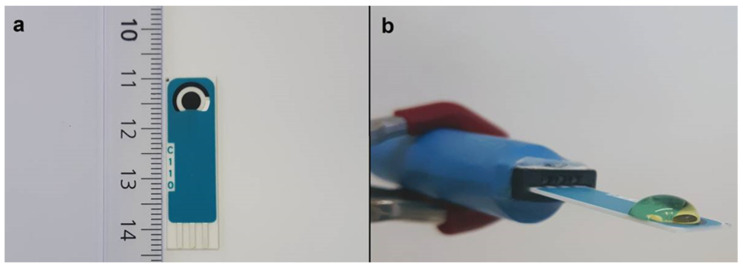
(**a**) Image of the SPCE electrode used to prepare the SOx/MXene–chitosan/SPCE biosensor. (**b**) Image of SOx/MXene–chitosan/SPCE electrode taken during CV analysis performed in 100 µL droplet in the potential window from +0.1 V to −1.3 V vs. silver RE at a scan rate of 0.1 V·s^−1^ using 10 (blank) or 3 (sarcosine) scans.

In this work, MXene Ti_3_C_2_T_x_ was applied to the integration of sarcosine oxidase (SOx) onto SPCEs to fabricate a miniaturised, portable, cost-effective nanobiosensor for the ultrasensitive analysis of sarcosine as a PCa biomarker (Figure 1).

The Materials and Methods section is provided in the supporting information file.

## 2. Results

### 2.1. Basic CV Studies in a Plain Buffer

**Figure 2:**
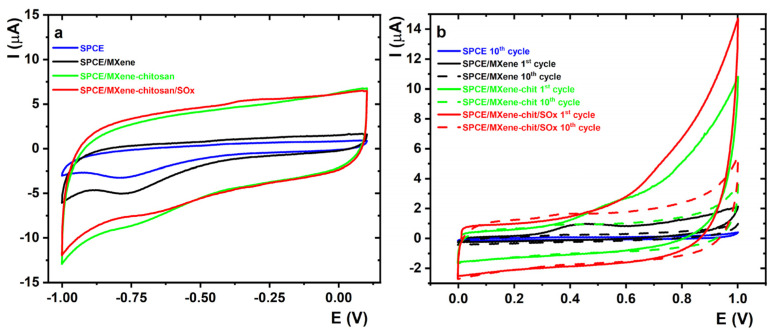
CVs in the plain 0.1 M PB pH 7.4 in (**a**) cathodic and (**b**) anodic potential windows with a scan rate of 100 mV·s^−1^ recorded using SPCE, MXene/SPCE, and MXene–chitosan/SPCE; SOx/MXene–chitosan/SPCE.

Initial electrochemical experiments were performed by running CVs in a plain 0.1 M PB pH 7.4 in potential windows from 0.1 to −1.0 V (a cathodic window) and from 0.0 to 1.0 V (an anodic window) with a scan rate of 0.1 V·s^−1^ using modified SPCEs. The following electrodes were examined: SPCE, MXene/SPCE, MXene–chitosan/SPCE, and SOx/MXene–chitosan/SPCE (Figure 2).

As shown in the Figure 2a, the capacitive current increased in the following order: unmodified SPCE, MXene/SPCE, MXene–chitosan/SPCE, and SOx/MXene–chitosan/SPCE. For MXene-modified SPCE, a higher current was observed than that of pristine SPCE because MXene/SPCE, due to the porous MXene structure, has a greater electrochemically active surface area when compared to the unmodified surface. These findings are in good agreement with already published results [35]. In the cases of MXene–chitosan/SPCE and SOx/MXene–chitosan/SPCE, the capacitive current was significantly higher than the unmodified SPCE and MXene/SPCE surface, suggesting the presence of enhanced MXene loading onto the electrode surface when present within the nanocomposite compared to the electrode modified only by MXene.

On the other hand, cyclic voltammetry in an anodic potential window (0.0 to 1.0 V) revealed that the MXene present on the MXene/SPCE surface underwent irreversible anodic oxidation with an oxidation peak at 0.457 V recorded in the first CV scan (9.67 ± 0.27) × 10^−7^ A (Figure 2b). The experiment revealed that the beneficial redox behaviour of MXene substantially dropped in the second scan, and a further decrease in anodic current with the increasing number of scans was observed. The exposure of MXene to an anodic potential in the aqueous solution oxidised the nanomaterial, forming a TiO_2_ layer or TiO_2_ domains with the subsequent TiO_2_ dissolution by F^–^ ions, rendering the resulting nanomaterial less electrochemically active compared to the pristine MXene [36]. Similar results could be observed in the cases of the MXene–chitosan/SPCE and SOx/MXene–chitosan/SPCE surfaces. The prepared devices could, thus, be applied to electrochemical reactions in a cathodic potential window.

### 2.2. EIS Measurements Using a Ferricyanide/Ferrocyanide Redox Couple

**Figure 3:**
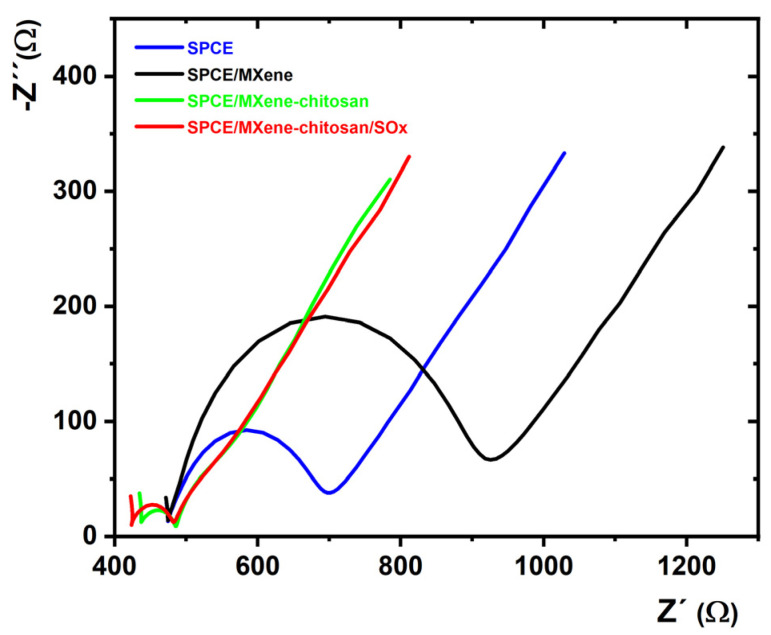
Nyquist plots of SPCE, MXene/SPCE, MXene–chitosan/SPCE, and SOx/MXene–chitosan/SPCE in 0.1 M PB containing 5 mM K_3_[Fe(CN)_6_] and K_4_[Fe(CN)_6_]·3H_2_O. Results are presented in the form of a Nyquist plot with equivalent circuit R[Q(RW)] applied for data fitting.

EIS is a powerful characterisation technique to probe the interfacial properties of individual unmodified and modified SPCEs. EIS measurements were performed to observe the characteristics of individual modified SPCEs, and investigate the short-term stability of the prepared MXene/SPCE and MXene–chitosan/SPCE surfaces. Figure 3 shows the impedance spectra of the prepared modified electrodes.

A Nyquist plot shows two main features: a semicircular part with a diameter representing charge transfer resistance R_ct_ and a linear part representing the diffusional properties of the interface [37].

EIS using a ferricyanide/ferrocyanide redox couple revealed that the R_ct_ of MXene-modified SPCE was approximately 1.9 times larger than that of bare SPCE (Table 1), indicating that a layer of negatively charged nanomaterial had formed on the surface, which hindered the electron transfer between [Fe(CN)_6_]^3−/4−^ and the electrode surface (Table 1). Though MXene could hinder the electron transfer of a negatively charged soluble redox probe pair, it is an excellent candidate with high metallic conductivity and great film-forming ability for the design of electrochemical biosensors. In contrast, the MXene–chitosan/SPCE and SOx/MXene–chitosan/SPCE surfaces exhibited significantly lower R_ct_ compared to those of the unmodified and MXene/SPCE. The R_ct_ value changed because of the electrostatic interactions between the positively charged chitosan-containing surface and [Fe(CN)_6_]^3−/4−^ ions.

MXenes are promising nanoplatforms for the construction of biosensing devices due to their excellent physicochemical properties. A natural biopolymer, chitosan, is an excellent counterpart to MXene since it is a hydrophilic hydrogel that can form adhesive membranes/films with biocompatible properties [38,39]. Thus, the combination of chitosan and nanomaterials such as MXene is an efficient strategy for the design of mechanically robust biosensing devices [40,41].

**Figure 4:**
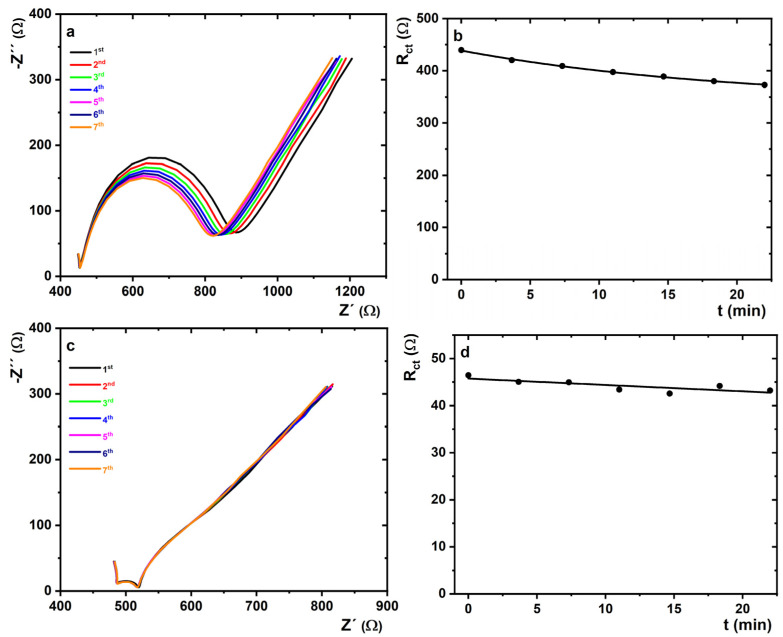
Nyquist plots of (**a**,**b**) MXene/SPCE and (**c**,**d**) MXene–chitosan/SPCE in 0.1 M PB pH 7.4 containing 5 mM K_3_[Fe(CN)_6_] and K_4_[Fe(CN)_6_]·3H_2_O with measurements repeated 7 times. Results are presented in the form of a Nyquist plot, with an equivalent circuit R[Q(RW)] applied for data fitting. The duration of the 7 subsequent EIS analyses was approximately 22 min.

In order to investigate the short-term stability of the prepared MXene/SPCE and MXene–chitosan/SPCE surfaces, and the stabilisation of nanomaterial with a chitosan matrix, seven subsequent EIS measurements were run in 0.1 M PB containing 5 mM K_3_[Fe(CN)_6_] and K_4_[Fe(CN)_6_]·3H_2_O at 50 different frequencies in the range from 0.1 Hz to 100 kHz. The obtained Nyquist plots of MXene/SPCE and MXene–chitosan/SPCE are presented in Figure 4.

The obtained results confirmed the stabilisation effect of the chitosan matrix on biosensor performance. In the case of the MXene/SPCE surface, a more significant change in R_ct_ value was observed compared to that on the MXene–chitosan/SPCE surface. The greatest decrease in R_ct_ value was recorded during the first four EIS measurements (Figure 4a). When the assay was performed in an electrochemical cell, a much quicker change (decrease) in R_ct_ was observed, i.e., 84.5% of the initial value on the MXene/SPCE interface (Figure 4b) compared to the assay performed on MXene–chitosan/SPCE (Figure 4c), i.e., 93.0% of the initial value (Figure 4d) within 22 min, indicating that chitosan had a positive effect on the stability of the MXene-modified interface. The exponential fitting of the operational stability curve performed on an electrochemical cell revealed that the signal would be stable at the R_ct_ value of 341 Ω (i.e., 77.6% of the initial value).

### 2.3. Detection of Sarcosine Using SOx/MXene–Chitosan/SPCE Biosensor

**Figure 5:**
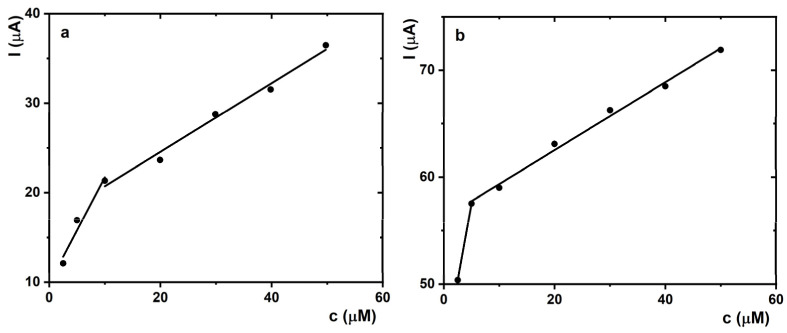
Calibration curves of the SOx/MXene–chitosan/SPCE biosensor vs. sarcosine concentration. Inset: CVs of the prepared SOx/MXene–chitosan/SPCE biosensor in 0.1 M PB pH 7.4 containing 0, 2.5, 5, 10, 20, 30, 40, 50 μM sarcosine at a scan rate of 0.1 V·s^−1^ in a potential window from +0.1 V to −1.0/−1.3 V. Measurements were run in the electrochemical cell by dipping SPCEs into (**a**) the solution or in (**b**) 100 μL of an electrolyte. CVs are shown in the Supplemental Figures S1 and S2, respectively.

CV measurements with SOx/MXene–chitosan/SPCE electrodes for detection of sarcosine were performed in a cathodic potential window. SOx enzyme was characterised by maximal stability at pH value of 7.4 [42]. The bioelectrochemical activity of the biosensor was evaluated by constructing the corresponding calibration curves when 10 mM sarcosine stock solution was added into the cell during the measurement. The final sarcosine concentration was in the range of 2.5–50 µM. Well-defined irreversible redox peaks were observed on the SOx/MXene–chitosan/SPCE electrode in the presence of sarcosine. The cathodic peak of H_2_O_2_ reduction increased with increasing biomarker concentration (Figure 5, inset). The plot of the peak current in the first CV scan vs. sarcosine concentration was applied to calibrate the device by subtracting a blank signal. In this work, blank was considered the fifth CV scan obtained in the absence of sarcosine (Figure 5).

In comparison with our previous study, several differences could be observed when working with SOx/MXene–chitosan/SPCE biosensor compared to SOx/MXene–chitosan/GCE device [43]. Significant differences in the values of the current responses were recorded. In the case of SPCE-based detection platform, they were about 19 times higher (1.21 × 10^−5^ A in the presence of 2.5 μM sarcosine) in comparison with those of GCE-based biosensing (6.38 × 10^−7^ A in the presence of 2.5 μM sarcosine). Changes in the current responses could be attributed to the properties of the carbon working-electrode material. The fitting of the calibration curve revealed two different ranges in which the calibration curve was linear for both types of measurements (Figure 5).

The assay in the electrochemical cell exhibited a first linear calibration curve in a range up to 10 μM with a slope of 1.19 ± 0.25 μA·μM^−1^ (R^2^ = 0.910), and a second linear calibration curve in the range of 10–50 μM with a slope of 0.38 ± 0.03 μA·μM^−1^ (R^2^ = 0.982) (Figure 5a). Additionally, the prepared SOx/MXene–chitosan/SPCE biosensor exhibited a satisfactory LOD value of 7.0 nM. Hence, the LOD was successfully reduced by 2.6 times [43]. In comparison with the SOx/MXene–chitosan/GCE device, a noticeable shift in peak potential values with respect to Ag/AgCl/KCl (3 M) RE was recorded. The peak potential shifted to more positive values with the increasing concentration of sarcosine. The electrochemical reduction in H_2_O_2_ by the device started at −0.2 V, with the peak maximum present at approx. −0.6 V (Figure 5, inset).

The proposed biosensing detection platform was subsequently further optimised. For this purpose, electrochemical measurements focusing on sample volume reduction were performed. For each measurement, drops with a volume of 100 µL were applied onto the surface of the SPCE’s working electrode, since SPCEs are suitable for either working with microvolumes or dipping them into solutions. In this case, the electrochemical cell consisted of a modified carbon working electrode, carbon auxiliary electrode, and silver RE (Figure 5b). The assay run in 100 μL of an electrolyte showed a first linear calibration curve in the concentration window up to 5 μM with a slope of 2.86 μA·μM^−1^ and a second linear calibration curve in the range of 5–50 μM with a slope of 0.32 ± 0.01 μA·μM^−1^ (R^2^ = 0.992) (Figure 5b). In comparison with the previous measurement (Figure 5a), a shift in the reduction process towards more negative potentials was observed. The electrochemical reduction in H_2_O_2_ started at −0.5 V with the peak maximum present at −0.9 V. Such an electrochemical behaviour can be assigned to the characteristics of the silver reference electrode used in three-electrode system. Moreover, similar findings were observed in the peak potential shift towards more positive values with an increasing concentration of sarcosine when compared to that in Figure 5a. Additionally, the prepared SOx/MXene–chitosan/SPCE biosensor exhibited a satisfactory LOD value of 10.4 nM. A slight increase in the LOD value in the case of the detection of sarcosine in the drop using the SOx/MXene–chitosan/SPCE device was caused by the increased intensity of noise during the electrochemical measurements. The intensified noise could be attributed to the nature of the used SPCE devices.

The reproducibility of the biosensor device’s fabrication, examined as the sensitivity of the devices, was within 10 % (*n* = 5) when measured in an electrochemical cell, and within 3 % (*n* = 4) when measured in 100 μL of an electrolyte. Such observations indicate that it is much better to use the biosensor device for measurements in 100 μL of an electrolyte with the bionanocomposite at the bottom in the measurement setup. Part of the bionanocomposite in the electrochemical cell was most likely dissolving over time.

The above MXene-based sarcosine biosensor device based on SPCEs is among the most sensitive applied to the analysis of sarcosine when comparing the LOD with that of other publications focused on the design of sarcosine sensors or biosensors (Table 2). Additionally, the as-fabricated biosensor belongs to the most sensitive MXene-based devices published so far when considering LOD (Table 3).

**Figure 6:**
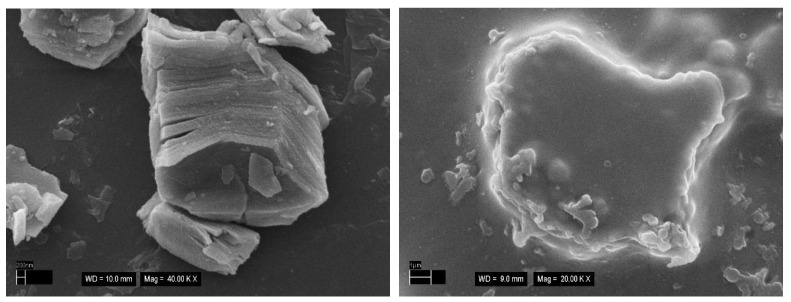
Scanning electron microscopy images representing (**left**) pristine multilayered Ti_3_C_2_T_X_ MXene structure, 40,000× and (**right**) Ti_3_C_2_T_X_ MXene@chitosan composite, 20,000×.

Scanning electron microscopy was used to visualise both pristine MXene (Figure 6, left) and the Ti_3_C_2_T_X_ MXene@chitosan composite (Figure 6, right).

### 2.4. Clinical Application of SOx/MXene–Chitosan/SPCE Biosensor

The practical application of the biosensor was evaluated by analysing the analyte in real samples. For that purpose, the SOx/MXene–chitosan/SPCE device was applied to analyse sarcosine (from 0.1 to 1.0 µM) spiked into 10× diluted artificial urine using sarcosine as the PCa biomarker [20]. Calibration curves were obtained from measurements in 0.1 M PB pH 7.4 and 10× diluted artificial urine together with the CVs of the prepared SOx/MXene–chitosan/SPCE biosensor. The value of the obtained recovery index was 92.5% (data not shown). The recovery index was calculated via the standard addition of 1 μM sarcosine into artificial urine, and the current response obtained in urine was compared to the current response obtained in plain PB. This means that, under such conditions, there were no significant interferences affecting the biosensor’s performance. The results prove that the as-fabricated biosensor is a reliable tool for sarcosine detection in urine samples. Obviously, analysis with real human urine is needed to reach more general conclusions about the performance of the prepared MXene-based sarcosine biosensor in the analysis of real urine samples.

### 2.5. Long-Term Stability of the SOx/MXene–Chitosan/SPCE Device

**Figure 7:**
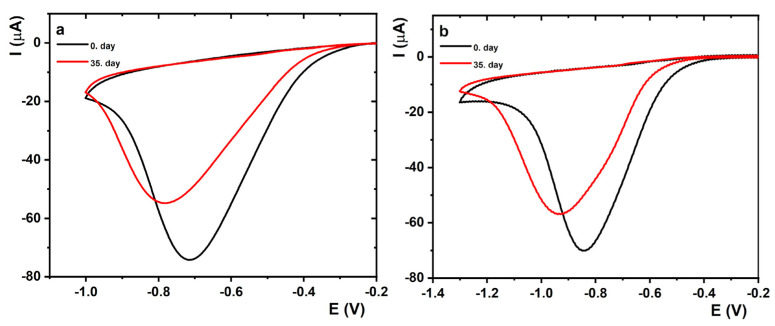
(**a**) CVs after the subtraction of the blank of the prepared SOx/MXene–chitosan/SPCE biosensor in 0.1 M PB pH 7.4 in a cell containing 30 μM sarcosine at a scan rate of 0.1 V·s^−1^ in a potential window from 0.1 to −1.0 V measured on days 0 (black) and 35 (red) after its preparation. (**b**) CVs after the subtraction of the blank of the SOx/MXene–chitosan/SPCE biosensor in 0.1 M PB pH 7.4 containing 30 μM sarcosine at a scan rate of 0.1 V s^−1^ in a potential window from 0.1 to −1.3 V measured on days 0 (black) and 35 (red) after preparation. During each measurement, 100 µL was applied to the surface of SPCE electrode.

To investigate the long-term stability of the prepared SOx/MXene–chitosan/SPCE device, CV measurements in the presence of 30 µM sarcosine in the potential window from 0.1 to −1.0 V and 0.1 to −1.3 V in the cell and in a 100 µL droplet, respectively, were performed (Figure 7). All prepared devices were stored in the refrigerator throughout the experiment.

In the case of the performed measurements in the cell (Figure 7a), the average current responses of the prepared SOx/MXene–chitosan/SPCE device decreased from 7.05 ± 0.51 × 10^−5^ A on day 0 to 5.94 ± 0.65 × 10^−5^ A on Day 35 after biosensor preparation. Thus, a 15.7% drop of the SOx/MXene–chitosan/SPCE current response was observed for measurements in the cell within 35 days. For the measurements performed in the 100 µL droplet (Figure 7b), a 6.8% decrease in the SOx/MXene–chitosan/SPCE current response was observed on Day 35 after fabrication. In this case, the average current responses of the prepared SOx/MXene–chitosan/SPCE device decreased from 6.12 ± 0.77 × 10^−5^ A on Day 0 to 5.71 ± 0.01 × 10^−5^ A on Day 35. Despite a slight decrease in the current response, the as-fabricated SOx/MXene-chitosan/SPCE device was able to detect sarcosine even after a period of 5 weeks. In summary, the obtained data confirm that the as-fabricated device could be used for analysis for at least 5 weeks after its preparation.

## 3. Conclusions

The goal of this paper was to design an enzymatic nanobiosensor for the detection of PCa biomarkers using disposable SPCEs and modern 2D nanomaterial MXene in order to prepare a sensitive, reliable, portable and miniaturised, stable, cost-effective, and quick detection platform based on previously obtained results and findings. By dipping SPCEs into the solution, the prepared SOx/MXene–chitosan/SPCE biosensor exhibited a satisfactory LOD value of 7.0 nM. Hence, the LOD was successfully reduced 2.6 times when compared to our previous paper. The proposed detection platform was subsequently further optimised with a focus on a sample volume reduction of up to 100 µL, exhibiting a satisfactory LOD value of 10.4 nM. To investigate the long-term stability of the prepared SOx/MXene–chitosan/SPCE device, CV measurements were performed in the presence of 30 µM in both the cell and a 100 µL droplet. For the measurements performed in the 100 µL droplet, only a 6.8% decrease in the SOx/MXene–chitosan/SPCE current response could be observed during the first 35 days after fabrication. Thus, the obtained data confirm that the as-fabricated device could be used for the detection of sarcosine in urine for a period of at least 5 weeks after its preparation. Moreover, to evaluate the potential application of the biosensor for the analysis of real samples, the as-fabricated device was successfully applied for the determination of sarcosine spiked into 10× diluted artificial urine with the 92.5% recovery index value. Such results prove that the as-fabricated biosensor is a reliable tool for sarcosine detection in urine samples. The portable SOx/MXene–chitosan/SPCE device is one of the most sensitive electrochemical biosensor devices for the analysis of sarcosine when classified by LOD. Additionally, the as-fabricated MXene-based biosensor is one of the most sensitive MXene-based devices.

## Figures and Tables

**Table 1:** R_ct_ values for each electrode modification obtained by running EIS in 0.1 M PB containing 5 mM K_3_[Fe(CN)_6_] and K_4_[Fe(CN)_6_]·3H_2_O at 50 different frequencies in the range from 0.1 Hz to 100 kHz.

SPCE Modification	Charge Transfer Resistance (R_ct_) Values (Ω)
SPCE	233 ± 4
MXene/SPCE	447 ± 10
MXene–chitosan/SPCE	52 ± 8
SOx/MXene–chitosan/SPCE	66 ± 1

**Table 2:** Basic operational parameters of various (bio)sensors for the detection of sarcosine.

Detection	Surface Modification	LOD (nM)	Linear Range (µM)	RT (s)	Stability	Application	Ref.
Amperometric	PVA–Ag/AuNPs–pphTEOS–SOx/GCE	500	0.5–7.5	17	NR	Aqueous media	[44]
Amperometric	SOx/EDC/NHS/Au/ZnONPs/SPEs	16	0.01–0.1	NR	60 days	Synthetic urine	[45]
Amperometric	SOx/CHIT/CuNPs/cMWCNT/AuE	0.0001	0.1–100	2	180 days	Human serum	[46]
Amperometric	SOxNPs/AuE	10	0.1–100	2	180	Urine	[47]
Amperometric	SOx/Pt@ZIF8/GCE	1060	5–30	NR	3	Urine	[48]
Amperometric	Nafion–SOx/Pt/AAO	50	0.05–100	NR	NR	Aqueous media	[20]
Amperometric	SOx/Pt/OIHMMP/GCE	130	1–70	NR	NR	Human serum	[49]
Amperometric	SOx/PAA/GCE	0.4	0.001–0.05	NR	15 days	Urine	[50]
Amperometric	SOx/Pt–Fe_3_O_4_@C/GCE	430	0.5–60	NR	NR	Human serum	[51]
Amperometric	Fe_3_O_4_@ZIF–8@MIP/AuE	0.0004	0.000001–0.0001	NR	NR	Urine	[52]
Amperometric	SOx/chitosan/Ti_3_C_2_T_X_/GCE	18	0.036–7.8	2	NR	Synthetic urine	[43]
Amperometric	SOx/chitosan/Ti_3_C_2_T_X_/SPE	7	0.1–1.0	NR	NR	Synthetic urine	this work
Potentiometric	MIP-based sensor	0.14	0.001–10	<120	>5 months	Aqueous media	[53]
Potentiometric	Antisarcosine–Ab–GFOX@graphite–powder@dibutyl phthalate-electrode	0.003	0.01–100	60	3–4 months	Aqueous media	[54]
Potentiometric	Antisarcosine–Ab@graphite–powder@dibutyl phthalate–electrode	0.005	0.001–10	60	3–4 months	Aqueous media	[54]
Impedimetric	MIP/AuNPs/SPCE	8.5	0.011–17.9	NR	~7 days	Aqueous media	[55]
Colorimetric	PdNP-based sensing platform	5.0	0.01–50	NR	NR	Urine	[56]
Colorimetric	NQS/GO/GCE	730	6.2–26.3	NR	NR	Aqueous media	[57]
Fluorimetric	Nanomaghemite/AuNPs/QD/peptide	0.05	0.005–0.05	NR	NR	Urine Cell lines	[58]
Fluorimetric	ssDNA aptamer-based sensor	55	0.1–2	NR	NR	Urine	[59]

Abbreviations: LOD—limit of detection, RT—response time, PVA—polyvinyl alcohol, pph-TEOS—partially prehydrolysed tetraethyl orthosilicate, CHIT—chitosan, cMWCNT—carboxylated multi-walled carbon nanotubes, Pt@ZIF8—nanoplatinum-loaded porous zeolitic imidazolate framework-8, AAO—anodised aluminium oxide, Pt/OIHMMP—platinum-supported mesoporous organic-inorganic hybrid molybdenum phosphonate, MIP—molecularly imprinted polymer, GO—graphene oxide, GO-based nanocomposite: Ab-GO@graphite-powder@dibutyl phthalate-electrode; NQS—1,2-naphthoquinone-4-sulphonic acid sodium salt, NR—not reported.

**Table 3:** Operational performance of electrochemical MXene-based (bio)sensors.

Analyte	Detection	LOD	Linear range	Reference
Glucose	Amperometry	5.9 µM	0.1–18 mM	[60]
H_2_O_2_	Amperometry	0.02 µM	0.1–260 µM	[61]
NO_2_^-^	Amperometry	0.12 µM	0.5 µM–11.8 mM	[35]
H_2_O_2_	Amperometry	14.0 nM	0.1–380 µM	[62]
H_2_O_2_	Voltammetry (DPV)	1.95 µM	2 µM–1 mM	[63]
H_2_O_2_	Amperometry	448 nM	490 µM–53.6 mM	[64]
AA,	Voltammetry (DPV)	0.25 µM,	Up to 750 µM	[64]
DA,		0.26 µM,		
UA,		0.12 µM,		
APAP		0.13 µM		
H_2_O_2_	Chronoamperometry	0.7 nM	NR	[36]
DA	FET	100 × 10^−9^ M	100 × 10^−9^–50 × 10^−6^ M	[65]
P53 gene	ECL	5 nM	10 nM–1 mM	[66]
Phenol	Amperometry	12 nM	0.05–15.5 µM	[67]
Cd^2+^,	Voltammetry (SWASV)	98 nM,	0.1–1.5 µM	[68]
Pb^2+^,		41 nM,		
Cu^2+^,		32 nM,		
Hg^2+^		130 nM		
BrO_3_^−^	Voltammetry	41 nM	50 nM–5 µM	[69]
Malathion	Voltammetry (DPV)	0.3 × 10^−14^ M	1 × 10^−14^–1 × 10^−8^ M	[70]
Sarcosine	Chronoamperometry	18 nM	36 nM–7.8 µM	[43]
Sarcosine	Cyclic voltammetry (CV)	7 nM	0.1–1.0 µM	This work

Abbreviations: AA—ascorbic acid, APAP—acetaminophen, DA—dopamine, DPV—differential pulse voltammetry, ECL—electrochemiluminescence, FET—field effect transistor, SWASV—square-wave stripping voltammetry, UA—uric acid [71].

## Data Availability

Research data are available upon request.

## Supplementary Materials

The following supporting information can be downloaded at: https://www.mdpi.com/article/10.3390/jfb14030161/s1, Figure S1: Enlarged graph shown in Figure 5A in the inset; Figure S2: Enlarged graph shown in Figure 5B in the inset.
