# Supplementary material for: Amperometric Miniaturised Portable Enzymatic Nanobiosensor for the Ultrasensitive Analysis of a Prostate Cancer Biomarker
Source: J Funct Biomater. 2023 Mar 17;14(3):161. doi: 10.3390/jfb14030161 (PMC10056543; doi:10.3390/jfb14030161)
Supplement: Supplementary file 1 [file jfb-14-00161-s001.zip › jfb-2261788-supplementary.pdf]

SUPPORTING INFORMATION

Article

Amperometric miniaturized portable enzymatic nanobiosensor for ultrasensitive analysis of prostate cancer biomarker

Stefania Hroncekova[1], Lenka Lorencova[1], Tomas Bertok[1], Michal Hires[1], Eduard Jane[1], Marek Bucko[1], Peter Kasak[2] and Jan Tkac[1]*

1 Institute of Chemistry, Slovak Academy of Sciences, Dubravska cesta 9, 845 38 Bratislava, Slovakia
2 Center for Advanced Materials, Qatar University, P. O. BOX 2713, Doha, Qatar
* Correspondence: Jan.Tkac@savba.sk

Materials and Methods

Materials

Sarcosine oxidase from *Bacillus* sp. (SOx, lyophilised powder, 25–50 U.mg^{-1}), sarcosine (N-methylglycine, $C_3H_7NO_2$, 98%), chitosan (medium molecular weight), acetic acid ($C_2H_4O_2$), potassium phosphate monobasic (KH_2PO_4) and potassium phosphate dibasic (K_2HPO_4) (1 M solutions for preparation of phosphate buffer - PB), Surine™ (a negative urine control for toxicology), potassium hexacyanoferrate (II) trihydrate ($K_4[Fe(CN)_6]\cdot 3H_2O$), potassium hexacyanoferrate (III) ($K_3[Fe(CN)_6]$) were obtained from Sigma-Aldrich. Zeba™ spin desalting columns MWCO 7k, 0.5 mL were received from Thermo Fischer Scientific. Ethanol (p.a, 96.3%) and potassium chloride (KCl, p.a.) were purchased from Slavus. $Ti_3C_2T_x$ MXene (termed as MXene in the following text) in the powder form was obtained from the Center for Advanced Materials, Qatar University (Doha, Qatar).

Methods

Preparation of $Ti_3C_2T_x$ MXene

MXene was prepared by procedure when hydrofluoric acid (HF) is formed *in-situ* by reaction of lithium fluoride (LiF) with hydrochloric acid (HCl) using a protocol described previously [35]. For each measurement, freshly prepared MXene dispersion was used. MXene dispersion was prepared by dispersing 3 mg of MXene powder in 1 mL of distilled water. In order to obtain homogeneous MXene dispersion, prepared solution (3 mg.mL^{-1}) was sonicated for 1 min.

Design of SOx/MXene-chitosan/SPCE biosensor

In all electrochemical measurements, SPCE (C110, Metrohm DropSens) were employed as working electrodes. SPCEs were washed with ethanol and subsequently dried using pure nitrogen stream *prior* to each electrochemical measurement. SOx/MXene-chitosan/SPCE biosensor was prepared using a previously described protocol [36]. *Prior* to electrode modification, 0.1% chitosan solution in 0.3% acetic acid and MXene dispersion (3 mg.mL^{-1} in distilled water) were mixed to obtain MXene concentration of 0.5 mg.mL$^{-1.}$ A mixture was shaken overnight at a temperature of 20 °C and at a rotation speed of 1,500 rpm to form a MXene-chitosan nanocomposite. Electrode surface was modified by casting of 20 μL of the above-prepared nanocomposite onto the surface of bare/pristine SPCEs. In the next step, SOx enzyme stock solution was desalted using Zeba Spin desalting columns and then diluted 4 times using 0.1 M PB pH 7.4. Finally, 20 μL of the diluted SOx enzyme solution was added onto modified SPCE and allowed to dry at room temperature in a laminar box. Such prepared SOx/MXene-chitosan/SPCE biosensor as well as individual SPCE, MXene/SPCE and MXene-chitosan/SPCE surfaces underwent electrochemical characterisations. To make a simplified description of the biosensor construction a layer by layer deposition process was applied by deposition of layers on SPCE in the following order: 1. MXene-chitosan and 2. Desalted SOx enzyme stock solution.

Electrochemical procedures

Electrochemical investigations were run on a Autolab PGSTAT 302N potentiostat/galvanostat having also an impedimetric module with (modified) SPCE used as a working electrode. For measurements in the electrochemical cell, an Ag/AgCl/KCl (3 M) reference electrode (RE) and a counter platinum electrode (Bioanalytical systems, USA) were used in a three-electrode cell system. For measurements in 100 μL droplet, silver reference electrode and counter carbon electrode were applied. All assays were run under Nova Software 1.10, and data acquired were evaluated using OriginPro 9.1.

Electrochemical techniques used for biosensor characterisation

Cyclic voltammetry (CV) measurements

In order to characterise electrochemical behaviour and performance, SPCE, MXene/SPCE, MXene-chitosan/SPCE, SOx/MXene-chitosan/SPCE electrodes were freshly prepared and electrochemically studied. Characterisation CV measurements were run in the electrochemical cell in the potential window from +0.1 V to -1.0 V (a cathodic potential window) and 0.0 V to +1.0 V (an anodic potential window) at a scan rate of 0.1 V.s–1 in 0.1 M PB pH 7.4 using 10 scans.

Electrochemical impedance spectroscopy (EIS) measurements

EIS measurements were run to observe characteristics of individual SPCE, MXene/SPCE, MXene-chitosan/SPCE, SOx/MXene-chitosan/SPCE interfacial layers using redox probes. Measurements were done using 0.1 M PB pH 7.4 containing 5 mM $K_3[Fe(CN)_6]$ and $K_4[Fe(CN)_6]\cdot 3H_2O$ as electrolytes. The EIS analysis was run in Faraday cage (Metrohm Autolab, Utrecht, Netherlands) at 50 different frequencies (ranged from 0.1 Hz to

100 kHz) at an open circuit potential (OCP) value. In order to investigate short-term stability of prepared MXene/SPCE and MXene-chitosan/SPCE surfaces, subsequent seven EIS measurements in 0.1 M PB pH 7.4 containing 5 mM $K_3[Fe(CN)_6]$ and $K_4[Fe(CN)_6]\cdot 3H_2O$ at 50 different frequencies in the range from 0.1 Hz to 100 kHz were performed. The duration of seven subsequent EIS analyses was approximately 22 min. The results were presented in a form of a Nyquist plot, with an equivalent circuit R[Q(RW)] applied for data fitting.

Electrochemical detection of sarcosine

For electrochemical determination of sarcosine, SOx/MXene-chitosan/SPCE electrodes (Fig. 1) were prepared and electrochemically studied. CV measurements were run in the presence of various concentrations of sarcosine (2.5 μM – 50 μM) either in the electrochemical cell or directly in 100 μL droplet (Fig. 1b). For measurements in cell, experiments were run in the potential window from +0.1 V to -1.0 V *vs.* Ag/AgCl/KCl (3 M). For measurements in 100 μL droplet, experiments were done in the potential window from +0.1 V to -1.3 V *vs.* silver RE. All CV measurements were run at a scan rate of 0.1 $V.s^{-1}$ using 10 (blank) or 3 (sarcosine) scans. Before CV measurements, all electrodes were conditioned by immersing into 0.1 M PB pH 7.4 for several minutes to remove residual components. Limit of detection was calculated as a signal-to-noise ratio (S/N) = 3.

Clinical application of SOx/MXene-chitosan/SPCE biosensor

Applied potential of the prepared SOx/MXene-chitosan/SPCE device was examined by evaluation of a recovery index (Eq. 1), CV measurement in the presence of clinically relevant concentrations of sarcosine (from 0.1 μM to 1.0 μM) in 10x diluted artificial urine (Surine™ negative urine control, Merck, Germany) in electrochemical cell in the potential window from +0.1 V to -1.0 V *vs.* Ag/AgCl/KCl (3 M) at a scan rate of 0.1 $V.s^{-1}$ using 3 scans (10 scans for blank) was performed.

$$re\ (\%) = \frac{slope_{surine}}{slope_{PB}} \times 100 \qquad \text{Equation 1}$$

where re is the value of recovery index (%), $slope_{surine}$ is slope value of calibration plots of the SOx/MXene-chitosan/SPCE biosensor obtained during measurements in 10 × diluted surine (artificial urine) and $slope_{PB}$ is slope value of calibration plots of the SOx/MXene-chitosan/SPCE biosensor obtained during measurements in 0.1 M PB pH 7.4.

Long-term stability of the SOx/MXene-chitosan/SPCE device

To investigate the long-term stability of prepared SOx/MXene-chitosan/SPCE device, CV measurements in the presence of 30 μM sarcosine in the potential window from +0.1 V to -1.0 V *vs.* Ag/AgCl/KCl (3 M) (+0.1 V to -1.3 V *vs.* silver RE respectively) both in the cell and in a 100 μL droplet at a scan rate of 0.1 $V.s^{-1}$ using 3 scans (10 scans for blank) were performed. Experiments were performed on day of biosensor preparation and then after 4; 7; 11; 14; 21; 28; and 35 days of storage at 4 °C.

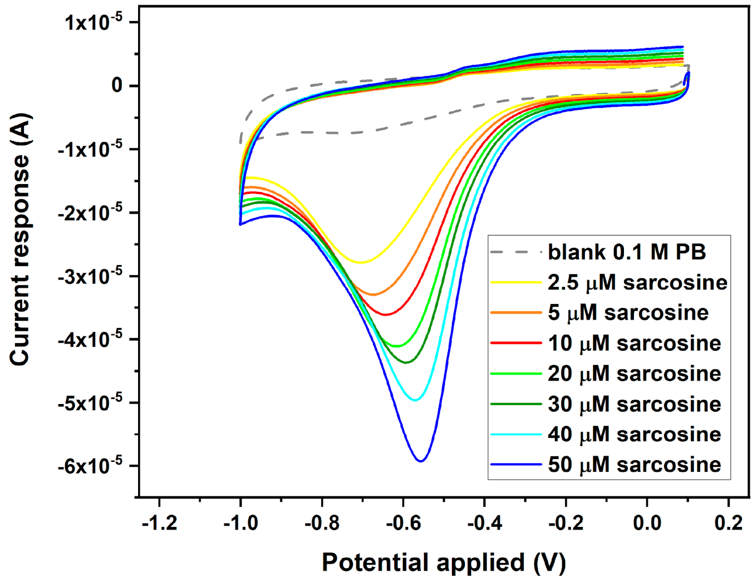

Figure S1: Enlarged graph shown in Fig. 5A in the inset.

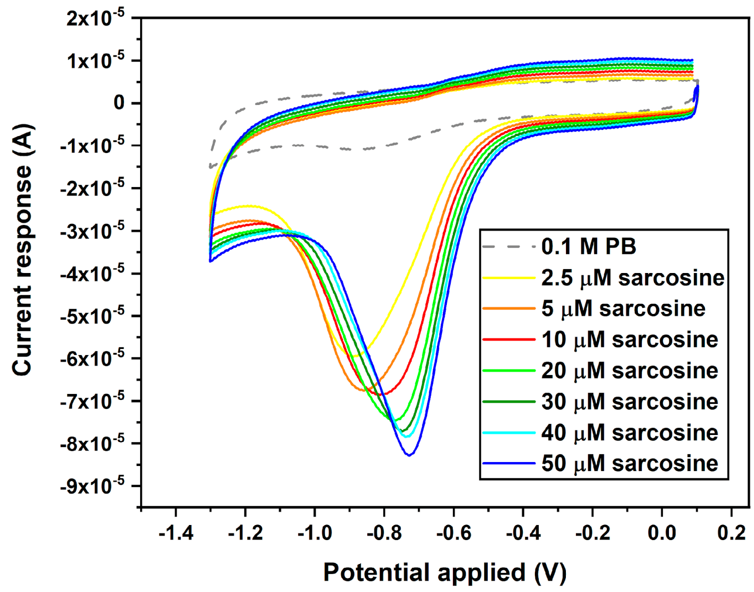

Figure S2: Enlarged graph shown in Fig. 5B in the inset.
